# Effects of Lifestyle Modification and Anti-diabetic Medicine on Prediabetes Progress: A Systematic Review and Meta-Analysis

**DOI:** 10.3389/fendo.2019.00455

**Published:** 2019-07-12

**Authors:** Zhi Sheng, Jia-Yu Cao, Ying-Chang Pang, Hang-Cheng Xu, Jing-Wen Chen, Jun-Hua Yuan, Rui Wang, Cai-Shun Zhang, Liu-Xin Wang, Jing Dong

**Affiliations:** ^1^Clinical Medicine Department, Medical College, Qingdao University, Shandong, China; ^2^Special Medicine Department, Medical College, Qingdao University, Shandong, China; ^3^Physiology Department, Medical College, Qingdao University, Shandong, China

**Keywords:** prediabetic state, drug therapy, healthy lifestyle, diabetes mellitus, type 2, randomized controlled trial, network meta-analysis, trial sequential analysis

## Abstract

**Background:** Pre-diabetes is a risk factor for full-blown diabetes; it presents opportunities to prevent the actual diseases. It is therefore essential to identify effective preventive strategies, and to clarify the direction of future research.

**Methods:** PubMed, Embase and Cochrane Central Register of Controlled Trials were searched using key terms (Supplementary Table 1). We applied network meta-analysis to multiple comparisons among various diabetic preventive strategies, including lifestyle and pharmacological interventions; traditional meta-analysis for the synthesis of basal metabolic changes after interventions; and trial sequential analysis for determinations as to whether analysis conclusions meet expectations.

**Results:** We included 32 randomized controlled trials comprising 43,669 patients and 14 interventions in the meta-analysis. Both lifestyle modifications and anti-diabetic medications improved physical conditions, including weight loss, blood glucose, and blood pressure. Network meta-analysis suggested that the progression of diabetes could be delayed to varying degrees by lifestyle and pharmacological interventions, except for angiotensin-converting enzyme inhibitors, statins, sulfonylureas and vitamin D. The risk ratios (RR) [95% credible interval (CrI)] compared with control were: GLP-1RAs 0.28 (0.15, 0.50), Orlistat 0.33 (0.18, 0.55), TZM 0.33 (0.16, 0.63), TZD 0.39 (0.27, 0.53), LST 0.54 (0.32, 0.88), lifestyle 0.58 (0.49, 0.67), LSM 0.62 (0.45, 0.80), GI 0.66 (0.46, 0.88), SU 0.67 (0.40, 1.00), Vitamin D 0.91 (0.59, 1.40), ACEI 0.93 (0.62, 1.40), statins 1.20 (0.84, 1.60).

**Conclusions:** In adults with pre-diabetes, firm evidence supports the notion that lifestyle modifications and metformin reduces the incidence of diabetes with an average of 20% relative risk reduction, while statins increase the relative risk 20%. We found that lifestyle modifications, promising long-term strategies involving three factors (nutrition, exercise, and weight loss) contribute to health by reducing BMI, body weight, waist and hip circumference, systolic and diastolic pressure, fasting, and 2-h postprandial blood glucose, total cholesterol and by increasing HDL. We made this determination using TSA, avoiding further waste of experimental resources.

## Introduction

Type 2 diabetes mellitus (hereafter referred to as diabetes) is a major health problem associated with excessive morbidity and mortality, affecting approximately 5% of adults worldwide with rapidly rising prevalence (1, 2). Pre-diabetes, the precursor stage of diabetes, includes impaired fasting glucose (IFG), and impaired glucose tolerance (IGT), characterized by fasting plasma glucose (PFG) ≥6.1 and <7.0 or 2-h plasma glucose (2hPG) ≥7.8 and <11.1 (3). There were recently introduced as risk factors for both diabetes and cardiovascular disease by the American Diabetes Association (4). As many as 5–10% of individuals with pre-diabetes develop diabetes each year (5), and approximately 70% of these will progress to diabetes during their lifetime (6). Fortunately, prevention of diabetes in the pre-diabetes stage can restore normal blood glucose levels (FPG <6.1, 2hPG <7.8), making early intervention crucial (7).

Guidelines from the American Diabetes Association suggest that individuals with pre-diabetes should undertake lifestyle modification to prevent the onset of diabetes, with healthy meals, increased physical exercise and weight reduction (8). Prescription medication has also been considered for the prediabetic population. Evidence supports that not only classic anti-diabetes drugs such as metformin and acarbose, but also newer agents such as GLP-1 receptor agonists help prevent the development of diabetes. Several randomized controlled trials (RCTs) diabetes-prevention strategies (lifestyle and/or pharmacological interventions) have been conducted (9, 10) and the literature has reviewed current achievements (11). Nevertheless, modern clinical therapies demand complex analyses for decision-making processes (12), in spite of traditional meta-analyses. Therefore, to assess physical outcomes of pre-diabetes interventions and to interpret the contemporary state of pre-diabetes research, we performed a Bayesian network meta-analysis and trial sequential analysis.

## Methods

### Search Strategies

The protocol of this review was registered in PROSPERO (ID: CRD 42018095121). Two review authors individually searched PubMed, Embase, Cochrane Central Register of Controlled Trials (CENTRAL) with database-appropriate terms and the text words (Supplementary Table 1). The reference lists of potentially relevant reviews were also screened. All references were eligible for inclusion regardless of language, published year and status.

### Inclusion Criteria

- RCTs published in peer-reviewed journals between 1/1/1965 and 1/5/2018;
- Patients were adults with pre-diabetes;
- Group allocation was based on lifestyle or medication interventions;
- Participants were randomly assigned;
- Cumulative duration of interventions and follow-ups had a minimum with 1 year;
- Objective results were available, including the incidence of diabetes, regression of pre-diabetes, and physical condition changes.

### Exclusion Criteria

- Medications that were forbidden in routine clinical practice, e.g., troglitazone (13) and phenformin (14) (Supplementary Table 3);
- With the compliance of American Diabetes Association guidelines, lifestyle modification standardized to include both adjusted healthy meals and increased physical exercise (Supplementary Table 2) to ensure homogeneity, e.g., a study that only included health education (15);
- Patients with a history of cardiovascular events and other diseases.

### Data Extraction and Quality Assessment

We extracted the incidence of diabetes, remission rate of pre-diabetes, and physical consequences with the principle of intention to treat analysis. Study quality was assessed using the Cochrane Collaboration's tool for risk bias (16).

### Statistical Analysis

Analyses were performed using Mantel-Haenszel and Bayesian random effects models using RevMan (version 3.4.3) and R software (version 3.4.4, www.r-project.org), respectively. The Cochran-Mantel-Haenszel test (CMH) was used in the analysis of stratified or matched categorical data, allowing an investigator to test the association between a binary predictor or treatment and a binary outcome. The Bayesian random effects model, a classical statistical methods of network meta-analysis, uses posterior probability to rank all the interventions involved in the comparisons and avoids the bias caused by repeated iteration in the estimation of parameters by frequency theory.

Consistent and simultaneous estimates of all interventions were obtained using Markov Chain Monte Carlo simulations using WinBugs software (version 1.4.3, http://www.mrc-bsu.cam.ac.uk/software/bugs/the-bugs-project-winbugs/). The results were recorded with RR and 95% credible interval (CrI). Trial sequential analysis (TSA, version 0.9.5.10 Beta, http://www.ctu.dk/tsa/downloads.aspx) was managed to evaluate the cumulative evidence according to the information size achieved to date. When the same studies were repeatedly observed, the probability of a Type 1 error increased. Therefore, trial sequential analysis was intended to evaluate the overall Type 1 error rate assured at the desired level. Furthermore, with the trial sequential analysis, a conclusion may sometimes be reached at a much earlier stage once significant results are observed, at consequently lower financial and/or human cost. The cumulative ranking plot and the surface under the cumulative ranking (SUCRA) helps the researcher make decisions. The values of SUCRA are between 0 and 1 (0 ≤ SUCRA ≤ 1). When SUCRA is 1, the intervention is absolutely valid, and when it is 0, the intervention is absolutely invalid.

## Results

### PRISMA Flow Diagram and Baseline

#### PRISMA Flow Diagram

**Figure 1:**
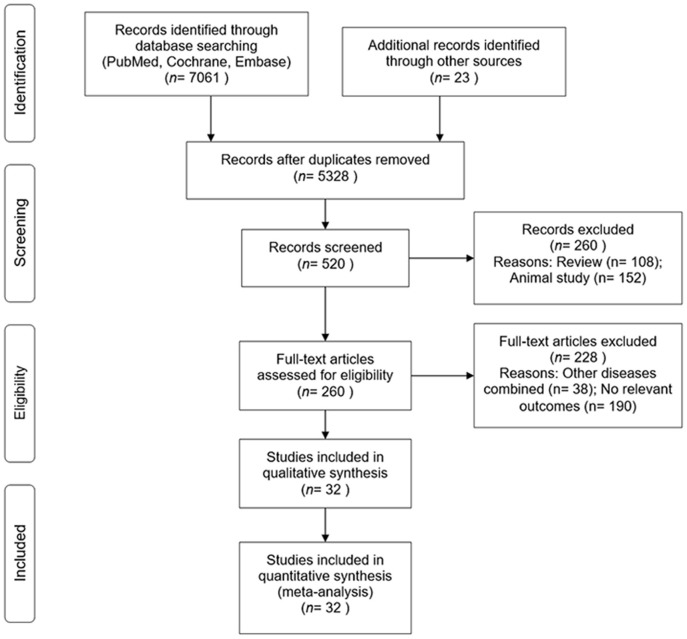
PRISMA flow diagram of selection of studies from search to final inclusion.

Results relating to identification and selection of eligible 32 RCTs with outcome data for the incidence of diabetes are summarized in Figure 1. These studies included 43,669 participants with a mean follow-up of 3.3 years ranging from 1 to 6 years.

#### Baseline Characteristics of Included Trials

The 32 RCTs included in the network meta-analysis are summarized in Table 1. In the network of available intervention comparisons, twelve trials focused solely on the effectiveness of lifestyle modification (all of these combined diet and exercise and health education was excluded), fourteen studies compared the effectiveness of only pharmacological interventions (nine anti-diabetic, two lipid lowering, one anti-obesity, one anti-hypertensive and one steroid), five studies combined lifestyle and pharmacological intervention groups and one combined the effect of the two medicines. No studies examined the effectiveness of surgical interventions.

**Table 1:** Baseline characteristics of included trials.

**Study**	**Year**	**Center (N)**	**Duration (y)**	**Inclusion criteria**	**Diagnosis^a^**	**Population^b^**	**Treatment**	**Diabetic incidence, *n/N* (%)**
ACT NOW (10)	2011	8	2.4	IGT	ADA 2008	Age: 52, 53	Placebo vs. pioglitazone (30–45 mg daily)	50/299 (17)
						Male (%): 42, 42		15/303 (5)
						BMI: 35, 33		
						Weight: NR		
CANOE (17)	2010	9	3.9^*^	IGT	WHO 1999	Age: 55, 50	Placebo vs. Rosiglitazone (2 mg) plus Metformin (500 mg) twice daily	41/104 (39)
						Male (%): 32, 35		14/103 (14)
						BMI: 32, 31		
						Weight: 86, 90		
Da Qing (18)	1997	33	6	IGT	WHO-	Age: 47, 44	Control vs. Lifestyle modification	90/133 (68)
						Male (%): 55, 56		58/126 (46)
						BMI: 26, 26		
						Weight: NR		
DPP (19)	2002	27	2.8	IFG/IGT	ADA 1997	Age: 50, 51, 51	Placebo vs. Metformin (850 mg twice daily) vs. Lifestyle modification	313/1082 (29)
						Male (%): 31, 34, 32		233/1073 (22)
						BMI: 34, 34 34		155/1079 (14)
						Weight:94, 94, 94		
DPS (9)	2001	5	3.2	IGT	WHO 1985	Age: 55, 55	Control vs. Lifestyle modification	59/257 (23)
						Male (%): 32, 34		27/265 (10)
						BMI: 31, 31		
						Weight: NR		
DREAM (20)	2006	191	3^*^	IGT, IFG	ADA 2003	Age: 55, 55	Placebo vs. Ramipril (5-15 mg daily)	489/2646 (18)
						Male (%): 40, 41		449/2623 (17)
						BMI: 31, 31		
						Weight: 85, 85		
DREAM (21)	2006	191	3^*^	IGT, IFG	ADA 2003	Age: 55, 55	Placebo vs. Rosiglitazone (8 mg daily)	658/2634 (25)
						Male (%): 40, 42		280/2635 (11)
						BMI: 31, 31		
						Weight: 85, 85		
EDIPS (22)	2009	6	3.1	IGT	WHO 1999	Age: 57, 57	Control vs. Lifestyle modification	11/ 51 (22)
						Male (%): 39, 41		5/ 51 (10)
						BMI: 34, 34		
						Weight: 91, 93		
Eriksson et al. (23)	2006	4	1.5	IGT	WHO 1999	Age: 53, 58	Placebo vs. Glipizide (2.5 mg daily)	5/17 (29)
						Male (%): 41, 12		1/17 (6)
						BMI: 29, 28		
						Weight: NR		
FDP (24)	2006	5	4^*^	IGT	WHO 1985	Age: NR	Control vs. Lifestyle modification	110/257 (43)
						Male (%): NR		75/265 (28)
						BMI: NR		
						Weight: 86, 87		
Heymsfield et al. (25)	2000	39	1.6	IGT	WHO 1980	Age: 44, 44	Placebo vs. Orlistat (120 mg 3 times daily)	4/53 (8)
						Male (%):16, 17		2/67 (3)
						BMI: 36, 36		
						Weight: 100, 99		
IDPP-1 (26)	2006	NR	3	IGT	WHO 1999	Age: 45, 46, 46, 46	Control vs. Lifestyle modification vs. Metformin (500 mg daily) vs. Lifestyle modification plus Metformin	75/136 (55)
						Male (%): 79, 76, 80, 81		52/133 (39)
						BMI: 26, 26, 26, 26		54/133 (41)
						Weight: NR		51/129 (40)
IDPP-2 (27)	2010	NR	3	IGT	WHO 1999	Age: 46, 46	Lifestyle modification vs. Lifestyle modification plus Pioglitazone (30 mg daily)	64/203 (32)
						Male (%): 84, 79		61/204 (30)
						BMI: 26, 26		
						Weight: NR		
Jorde et al. (28)	2016	1	5	IFG, IGT, IFG/IGT	WHO 1999	Age: 62, 62	Placebo vs. Vitamin D (20,000 IU per week)	112/255 (44)
						Male (%): 60, 63		103/256 (40)
						BMI: 30, 30		
						Weight: NR		
JUPITER (29)	2012	4	5	MS or IFG or BMI≥30 or HbA1c>6%	NR	Age: 66	Placebo vs. Rosuvastatin (20 mg daily)	204/5765 (4)
						Male (%): 59		258/5743 (4)
						BMI: 31		
						Weight: NR		
Kawamori et al. (30)	2009	103	3	IGT	WHO 1999	Age: 56, 56	Placebo vs. Voglibose (0.2 mg 3 times daily)	84/883 (10)
						Male (%): 60, 60		40/897 (4)
						BMI: 26, 26		
						Weight: NR		
Kosaka et al. (31)	2005	NR	4	IGT	WHO 1980	Age: 51.5	Control vs. Lifestyle modification	32/356 (9)
						Male (%): NR		3/102 (3)
						BMI: 24, 24		
						Weight: NR		
Le Roux et al. (32)	2017	191	3	Prediabetes	ADA 2010	Age: 47, 48	Placebo vs. Liraglutide (3.0 mg daily)	46/749 (6)
						Male (%): 24, 24		26/1505 (2)
						BMI: 39, 39		
						Weight: 108, 108		
Li et al. (33)	1999	NR	1	IGT	WHO 1985	Age: 50, 49	Placebo vs. Metformin (250 mg 3 times daily)	6/43 (14)
						Male (%): 73, 70		3/42 (7)
						BMI: 26, 26		
						Weight: NR		
Liao et al. (34)	2002	NR	1.5	IGT	WHO 1999	Age: 52, 56	Control vs. Lifestyle modification	2/38 (5)
						Male (%): 53, 37		1/36 (3)
						BMI: 27, 26		
						Weight: 70, 66		
Lindahl et al. (35)	2009	4	5	IGT	WHO 1985	Age: 54, 52	Control vs. Lifestyle modification	63/150 (42)
						Male (%): 39, 30		34/151 (23)
						BMI: 30, 31		
						Weight: 84, 86		
Lindblad et al. (36)	2010	23	5	IFG	NR	Age: 60, 60	Placebo vs. glimepiride (1 mg once daily)	55/138 (40)
						Male (%): 75, 88		41/136 (30)
						BMI: 30, 30		
						Weight: NR		
LIPID (37)	2003	5	5	IFG	WHO 1999	Age: 63	Placebo vs. Pravastatin (40 mg daily)	43/466 (9)
						Male (%): 85		46/474(10)
						BMI: NR		
						Weight: NR		
Polanco et al. (38)	2015	NR	6	prediabetes	NR	Age: NR	Lifestyle modification vs. Lifestyle modification plus Metformin (850 mg twice daily)	19/50 (38)
						Male (%): NR		10/52 (19)
						BMI: NR		
						Weight: NR		
Saito et al. (39)	2011	38	3	IFG	ADA 2003	Age: 48, 50	Control vs. Lifestyle modification	51/330 (15)
						Male (%): 71, 72		35/ 11 (11)
						BMI: 27,27		
						Weight: 75, 74		
Sakane et al. (40)	2011	32	3	IFG, IGT	WHO 1985	Age: 30-60	Control vs. Lifestyle modification	18/152 (12)
						Male (%): NR		9/152 (6)
						BMI: 25, 25		
						Weight: 64, 65		
SLIM (41)	2008	1	3	IGT	WHO 1999	Age: 51, 51	Control vs. Lifestyle modification	18/73 (25)
						Male (%): 56, 54		8/74 (11)
						BMI: 29, 30		
						Weight: 83, 88		
STOP-NIDDM (42)	2002	23	3.3	IGT	WHO 1985	Age: 55, 54	Placebo vs. Acarbose (100 mg 3 times daily)	285/686 (42)
						Male (%): 50, 48		221/682 (32)
						BMI: 31, 31		
						Weight: 87, 88		
Weber et al. (43)	2016	1	3	IFG, IGT, IFG/IGT	ADA 1997	Age: 44,45	Placebo vs. Metformin (500 mg daily)	98/295 (33)
						Male (%): 63, 64		69/283 (24)
						BMI: 28, 28		
						Weight: 75, 75		
XENDOS (44)	2004	22	4	IGT	WHO 1994	Age: 44, 43	Lifestyle modification plus placebo vs. Lifestyle modification plus orlistat (120 mg daily)	99/344 (29)
						Male (%): 45, 45		66/350 (19)
						BMI: 37, 37		
						Weight: 110, 110		
Xu et al. (45)	2012	1	1	IGR	ADA 2003	Age: 54, 41	Control vs. Lifestyle modification	7/42 (17)
						Male (%): 45,45		6/46 (13)
						BMI: 27, 26		
						Weight: 70, 68		
Zong et al. (46)	2015	6	2	Prediabetes	NR	Age: NR	Control vs. Lifestyle modification	11/107 (10)
						Male (%): NR		3/107 (3)
						BMI: NR		
						Weight: NR		

*Date are mean (SD or range)*.

* *Median (range)*.

*2hPG, 2-h plasma glucose; ADA, American Diabetes Association; BMI, body mass index; FBG, fasting blood glucose; HbA1c, glycated hemoglobin; IFG, impaired fasting glucose; IGT, impaired glucose tolerance; NR, not reported; OGTT, oral glucose tolerance test; WHO, World Health Organization*.

a *WHO Criteria 1980: IGT: FBG <140 mg/dl and OGTT 140–199 mg/dl. WHO Criteria 1985: normal: not defined; IGT: FBG <140 mg/dL and OGTT 140-200 mg/dL; IFG: not defined. WHO Criteria 1994: Not Found. WHO Criteria 1999: normal: FBG <110 mg/dL; IGT: FBG <126 mg/dL and OGTT 140-200 mg/dL; IFG: FBG 110-126 mg/dL and OGTT <140 mg/dL. ADA Criteria 1997: normal: FBG <110 mg/dL and OGTT <140 mg/dL; IGT: OGTT 140 mg/dL-200 mg/dL, IFG: FBG 110−126 mg/dL. ADA Criteria 2003: normal: FBG <110 mg/dL and OGTT <140 mg/dL; IGT: OGTT 140 mg/dL-200 mg/dL, IFG: FBG100-124 mg/dL. ADA Criteria 2008: normal: FBG <100 mg/dL and OGTT <140 mg/dL; IGT: OGTT 140 mg/dL-199 mg/dL, IFG: FBG 100-125 mg/dL, Diabetes FBG > 100 mg/dL or OGTT > 200 mg/dL. ADA Criteria 2010: Prediabetes: HbA1c 5.7–6.4%, or FBG 5.6-6.9 mmol/L, or 2hPG 7.8-11.0 mmol/L. Diabetes: HbA1c > 6.5% or FBG > 7.0 mmol/L, or 2hPG > 11.1 mmol/L*.

b *Age is reported in years and weight is reported in kilograms*.

Inclusion criteria of the valid 32 RCTs includes IGT, IFG and IGR. The diagnosis consists of World Health Organization (1980, 1985, 1994 and 1999); American Diabetes Association (1997, 2003, 2008, 2010). Population characteristics, including age, gender, BMI, weight and other data are displayed in Table 1.

### Network Meta-Analysis

As opposed to those of previous studies, in our review, diabetic prevention strategies were divided into 14 groups comprising the considerable baseline of all included studies: Control (standard care or placebo), GI (α-glucosidase inhibitor), GLP-1RAs (glucagon-like peptide 1 receptor agonists), Lifestyle (lifestyle modification), LSM (lifestyle modification plus metformin), LST (lifestyle modification plus thiazolidinedione), Metformin (metformin), Orlistat (orlistat), Statins (statins), SU (sulfonylureas), TZD (thiazolidinedione), TZM (thiazolidinedione plus metformin) Vitamin D (vitamin D) and ACEI (angiotensin converting enzyme inhibitors).

**Figure 2:**
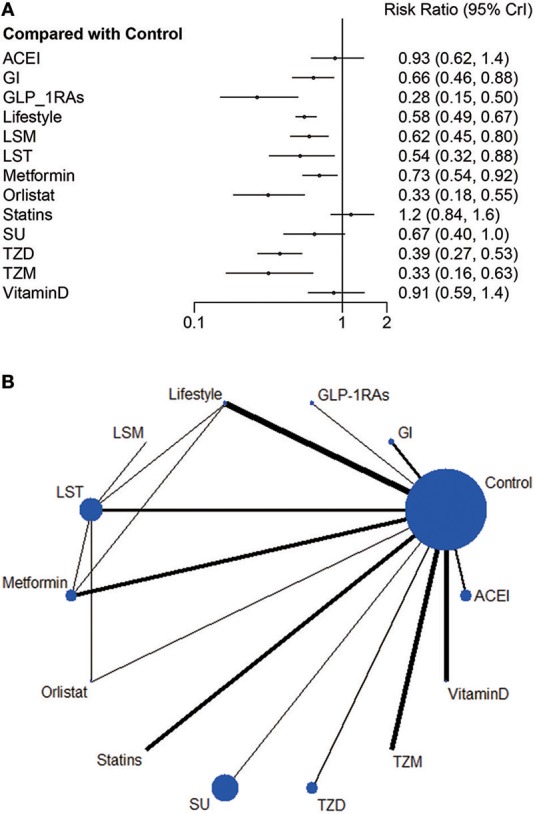
**(A)** Relative risk ratio and 95% credible interval of strategy interventions compared to control group in Bayesian random effect model of network meta-analysis. **(B)** Network plot: Weight the nodes according to the number of patients that have received each treatment; calculate the control group risk for studies including the control and weight the edges according to the mean control group risk for all comparisons vs. control.

Network meta-analysis uses indirect comparison technology to comprehensively evaluate and rank all interventions in a body of evidence. The network of all direct and indirect comparisons of all commonly using anti-pre-diabetes strategies can be seen in Figure 2B.

Compared with the control group, current evidence suggests that nine strategies credibly reduced incidence of diabetes with RR ranging from 0.28 (0.15, 0.50) to 0.73 (0.54, 0.92), including GLP-1RAs, Orlistat, TZM, TZD, LST, Lifestyle, LSM, SU and Metformin. Nevertheless, SU, ACEI, statins and Vitamin D all intersect the ineffective line (Figure 2A).

**Figure 3:**
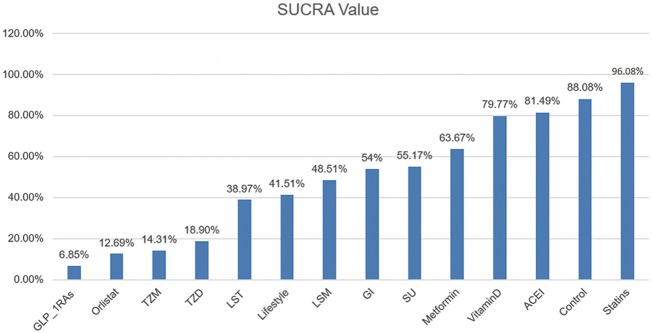
SUCRA Value of Diabetic Incidence. We ranked all fourteen intervention strategies based on their probabilities of prediabetes leading to diabetes and calculated SUCRA to obtain a more precise sorted consequence. The lower the SUCRA value, the more likely this measure is to prevent the progression of the diabetes process.

After comparing several interventions, the investigator is informed of the optimal intervention. However, if optimal intervention is not available, or is difficult to implement or expensive, researchers need to consider interventions beyond the optimal intervention. The order of interventions can be ranked according to the size of the SUCRA value. Because this article explores the adverse event rates of diabetes, the greater the value, the less prioritized is the intervention (Figure 3).

Consistent with the findings of previous studies (47), statins exposed participants to an incidence of diabetes greater than that of control and other intervention groups. The slight difference of LST, Lifestyle and LSM suggested a lack of evidence for supporting the superiority of lifestyle modification and these two pharmacological combination therapies.

The results of GI, LSM and SU (I^2^ = 72.5%, 48.2%, 70.6%) exhibited high heterogeneity vs. control (Supplementary Figure 1). We suspect that each of GI, SU, or LSM contains two or more medicine or lifestyle interventions, possibly explaining the higher heterogeneity.

### Physical Consequence of Interventions

**Figure 4:**
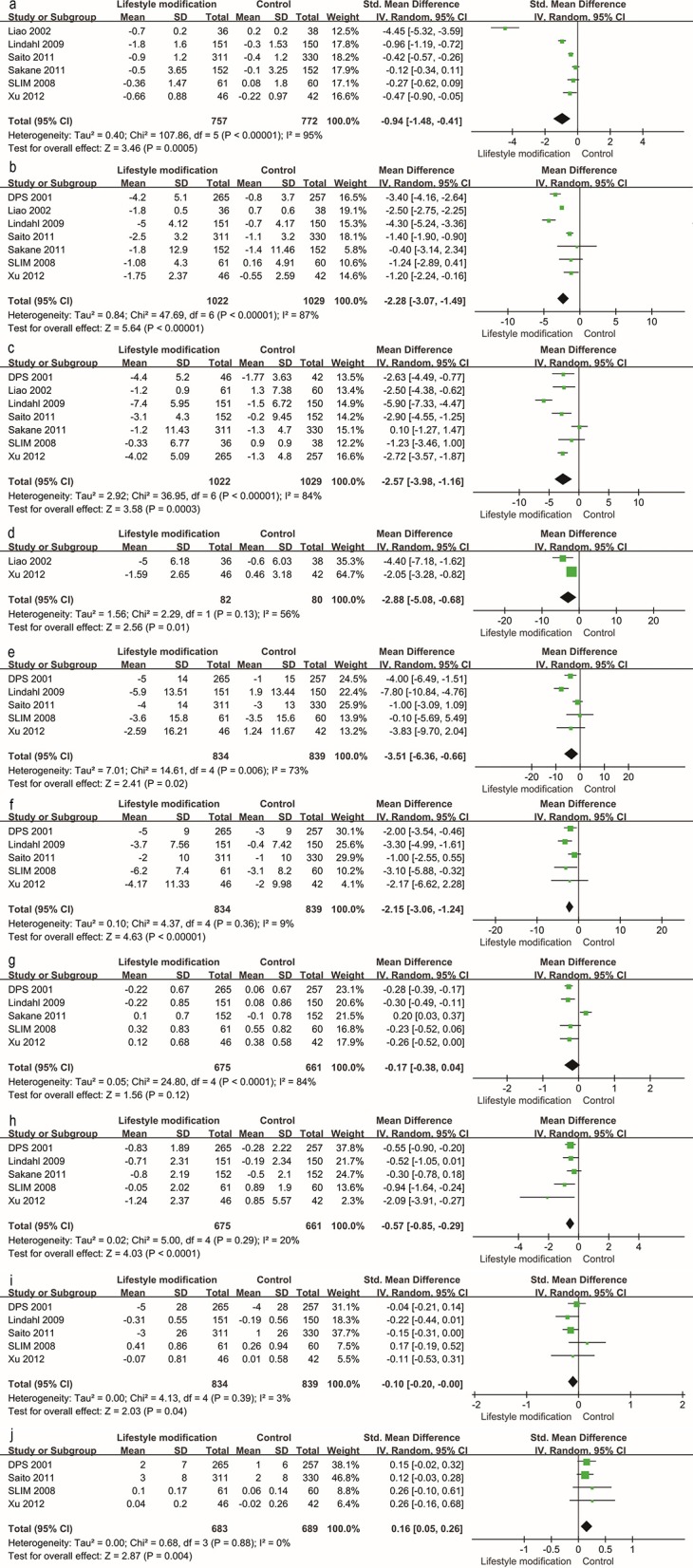
Traditional meta-analysis of the effect on physical conditions: Lifestyle modification vs. Control. **(a–j)** BMI (kg/m^2^), body weight (kg), waist circumference (cm), hip circumference (cm), systolic pressure (mmHg), diastolic pressure (mmHg), fasting blood glucose (mg/dL), 2 h postprandial blood glucose (mg/dL), total cholesterol (mmol/L, mg/dL), HDL (mmol/L, mg/dL).

**Figure 5:**
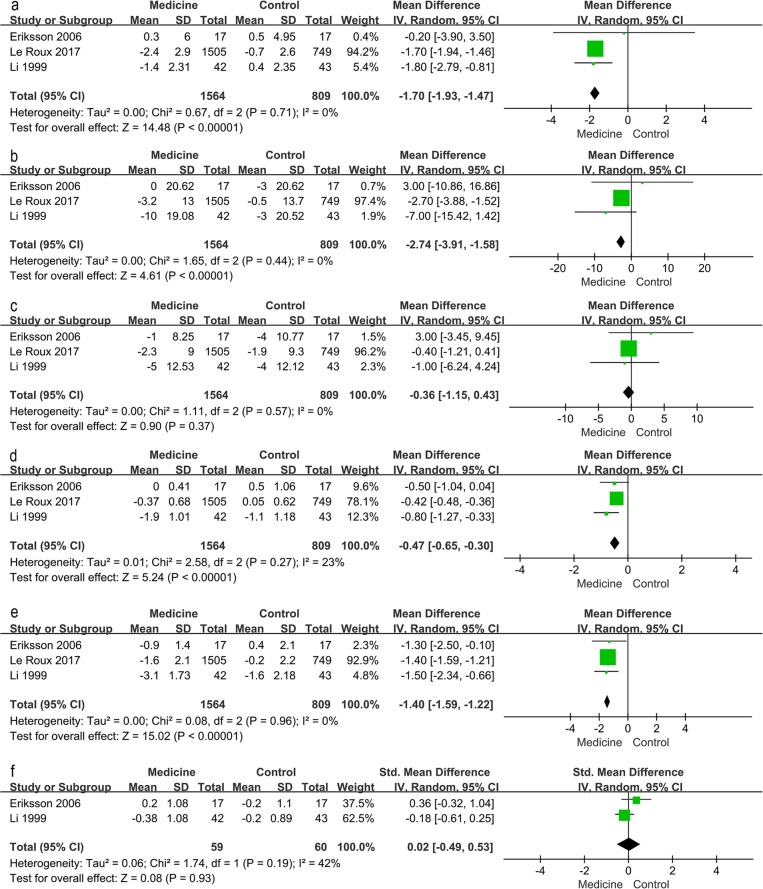
Traditional meta-analysis of the effect on physical conditions: Medicine vs. Control. **(a–f)** BMI (kg/m^2^), systolic pressure (mmHg), diastolic pressure (mmHg), fasting blood glucose (mg/dL), 2 h postprandial blood glucose (mg/dL), total cholesterol (mmol/L, mg/dL).

Traditional meta-analysis supported the benefits of both lifestyle modification and anti-diabetic medication. Lifestyle modification with a duration of at least 1 year decreases body mass index (BMI), body weight, waist and hip circumference, systolic and diastolic blood pressure, 2-h postprandial blood glucose, and increases serum HDL (Figure 4). BMI, body weight, waist and hip circumference, systolic pressure and fasting blood glucose exhibited high heterogeneity (I^2^ value exceeded 50%, I^2^ = 95, 87, 84, 56, 73, and 84% respectively); however, all these individual trials supported physical improvements except for fasting blood glucose. Anti-diabetic medication (including GLP-1RAs and insulin-sensitizing agents) decreased BMI, systolic blood pressure, fasting blood glucose and 2-h postprandial blood glucose (Figure 5).

### Trial Sequential Analysis

**Figure 6:**
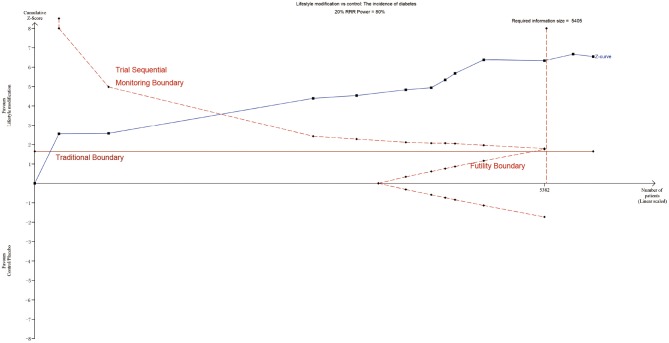
TSA of lifestyle modification. Effect of lifestyle modification vs. control on diabetes using a required information size of 5,405 participants in order to detect or reject a 20% RRR with a power of 80%.

TSA was performed to evaluate random errors caused by limited data and repetitive testing of accumulating data. The cumulative z-curve crossed both the traditional boundary and the trial sequential monitoring boundary but not the futility boundary, suggesting firm evidence for an average of 20% relative risk reduction of diabetes with lifestyle modification (Figure 6). Similarly, TSA supported sufficient evidence for 20% relative increased risk of diabetes with statins and 20% relative risk reduction of diabetes with metformin (Supplementary Figure 2A). The lack of evidence for a 30%, 60% and 25% relative risk reduction in diabetes with GI, orlistat and sulfonylureas demands larger trials (Supplementary Figure 2B). Other intervention strategies failed to establish such an analysis for the limited information size.

### Credibility Analysis

**Figure 7:**
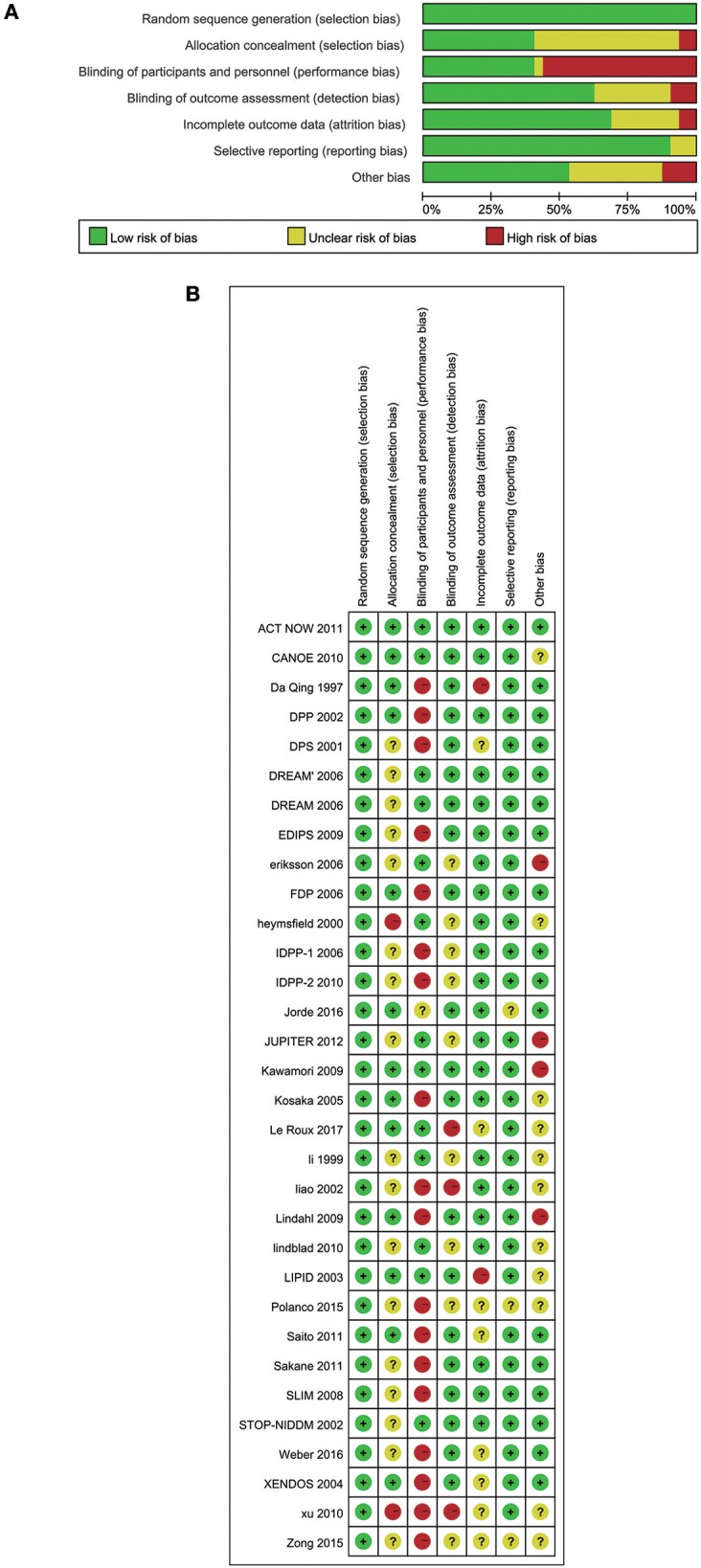
**(A)** Risk of bias graph: review authors' judgements about each risk of bias item presented as percentages across all included 32 studies. **(B)** Risk of bias summary: review authors' judgements about each risk of bias item for each included study.

We assessed several biases using Cochrane Collaboration's tool rating risk bias (Figures 7A,B). However, when trials assigned participants to undertake lifestyle modification, the potential allocation concealment were generated, increasing the likelihood of significant findings (48). Therefore, we should understand that the effects of lifestyle modification were at risk of exaggeration. Various definitions of the IFG, IGT, pre-diabetes and diabetes definitions in the trials may also interfere with the final results.

## Discussion

A total of 32 RCTs with available data contributed to this trial analysis, including traditional and network meta-analyses, TSA of the incidence of diabetes and a traditional meta-analysis of physical conditions.

Compared to placebo (Figure 2A), GLP-1RAs (0.28, [0.15, 0.50]), TZM (0.33, [0.16, 0.63]), and TZD (0.39, [0.27, 0.53]) significantly delayed the progression of diabetes; however, the limited sample size and the small quantity of studies caused instability of this inference. The data of both GLP-1RAs and orlistat were captured from severely obese people (mean BMI = 39 and 37 respectively), contributing to potential inconsistency. Metformin is less effective in people with lower baseline BMIs or lower FPG concentrations than in those with higher values for these variables; the drug works by inhibiting endogenous glucose production (49). It is not as flexible as lifestyle modifications that can be adjusted according to the specific physical conditions of the individual. Several studies reported that vitamin D supplementation reduced the incidence of diabetes in patients with both pre-diabetes and vitamin D deficiency (50); however, our review, similar to Angellotti and Pittas (51) showed the controversial result that vitamin D did not protect the prediabetic population without vitamin D deficiency from developing diabetes. There is evidence suggesting that obesity patients are susceptible to GLP-1RAs (32) and orlistat (25) on the progression of diabetic prevention. For population with prediabetes and other metabolic disturbances, including higher body weight or blood pressure or dyslipidemia, lifestyle modification should be a considerable intervening measure. Current researches support that patients are expected to benefit from GI (42) and statins for cardiovascular risk reduction (29, 37).

According to a review of collected trials, Haw et al. (11) suggested that lifestyle modification was a promising long-term diabetes prevention strategy; nevertheless, its sustained protective effects relied on maintenance interventions, even intermittent ones. This was consistent with prior results, to the effect that lifestyle interventions can somewhat prevent the conversion of pre-diabetes into diabetes. TSA can verify type I errors, thus avoiding more experiments to re-confirm this result, resulting in a waste of resources. Furthermore, reductions in BMI, body weight, waist and hip circumference induced by lifestyle intervention are expected to improve individual physical conditions, because weight loss appears to be the key factor associated with reduced diabetes progression (11). Their findings supported the use of pharmacological interventions (weight loss and insulin-sensitizing agents) to reduce diabetes incidence, and when the drug is eliminated from the body, its therapeutic effect will be weakened or even disappeared. It was suggested that the differences in insulin sensitivity and insulin secretion between IGT and IFG, and the greater severity of the abnormalities when both coexist might predict different rates of progression to diabetes, and different pharmacological agents might be needed to treat the pathophysiology. Recently, Pang et al. (52) reported multiple-treatment comparisons to discuss various diabetes preventing strategies in China, filling an investigative gap in traditional Chinese medicine. For the first time, we summarized the previous overview of pre-diabetes studies, and have found that medications and lifestyle interventions improve individual physical metabolism variously, permitting caregivers to individualize preventive care appropriate to individual clinical characteristics. The associated risk reduction of lifestyle modification, including healthy meals, increased physical exercise and weight loss is more pronounced than the effect of any single factor alone.

In order to distinguish unfinished and completed conclusions, avoid exceeding experimental waste of resources and therefore guiding the next step of clinical research, the collected studies were tested using TSA.

Despite the fact that this review performed trial sequential analysis of intervention strategies, the diabetes incidence was reported only once for ACEI, GLP-1RAs, LST, TZM and vitamin D, creating potential bias. Complications of diabetes increase patient suffering and mortality. Effective interventions may also delay or prevent complications, thereby significantly reducing the personal and public health burden of diabetes. Therefore, more relevant trials are needed to reinforce or further complement this review, especially for endpoints of clinical complications, such as cardiovascular events/death and data on cost-effectiveness.

## Conclusions

In adults with pre-diabetes, firm evidence supports the notion that lifestyle modifications and metformin reduces the incidence of diabetes with an average of 20% relative risk reduction, while statins increase the relative risk 20%. We found that lifestyle modifications, promising long-term strategies involving three factors (nutrition, exercise and weight loss) contribute to health by reducing BMI, body weight, waist and hip circumference, systolic and diastolic pressure, fasting and 2-h postprandial blood glucose, total cholesterol and by increasing HDL. We made this determination using TSA, avoiding further waste of experimental resources.

## Consent For Publication

The corresponding author had final responsibility for the decision to submit for publication.

## Author Contributions

ZS and J-YC contributed equally to this work, including the conception and design research, data extraction, data analysis, and drafted the composition. Y-CP, H-CX, and J-WC contributed to statistical analysis. J-HY, RW, C-SZ, and L-XW conducted the proofreading work. JD contributed to crucial revisal of the treatise for important intellectual content.

### Conflict of Interest Statement

The authors declare that the research was conducted in the absence of any commercial or financial relationships that could be construed as a potential conflict of interest.

## Supplementary Material

Supplementary Figure 1 Heterogeneity test in network meta-analysis using I2 in pair-wise and network pooled comparison.

Supplementary Figure 2 **(A)** TSA of metformin and statins. Effect of metformin vs. control on diabetes using a required information size of 3,388 participants in order to detect or reject a 20% RRR with a power of 80%; effect of statins vs. control on diabetes using a required information size of 15,632 participants in order to detect or reject a 20% relative increased risk with a power of 80%. **(B)** TSA of GI, orlistat and sulphonylureas. Effect of GI vs. control on diabetes using a required information size of 11,993 participants in order to detect or reject a 30% RRR with a power of 80%; effect of orlistat vs. control on diabetes using a required information size of 368 participants in order to detect or reject a 60% RRR with a power of 80%; effect of sulphonylureas vs. control on diabetes using a required information size of 1,003 participants in order to detect or reject a 25% RRR with a power of 80%.

Supplementary Table 1 Database-appropriate terms and the text words.

Supplementary Table 2 Description of lifestyle modification.

Supplementary Table 3 Baseline characteristics of excluded trials.

## Abbreviations

ACEI: Angiotensin converting enzyme inhibitors
BMI: Body mass index
CENTRAL: Cochrane Central Register of Controlled Trials
CrI: Credible interval
GI: α-glycosidase inhibitors
GLP-1RAs: Glucagon-like peptide 1 receptor agonists
LSM: Lifestyle modification plus metformin
LST: Lifestyle modification plus thiazolidinedione
RCT: Randomized controlled trials
SUCRA: Surface under the cumulative ranking probabilities
TZD: Thiazolidinedione
TZM: Thiazolidinedione plus metformin.
