# Supplementary figures and images for: Effects of Lifestyle Modification and Anti-diabetic Medicine on Prediabetes Progress: A Systematic Review and Meta-Analysis
Source: Front Endocrinol (Lausanne). 2019 Jul 12;10:455. doi: 10.3389/fendo.2019.00455 (PMC6639788; doi:10.3389/fendo.2019.00455)

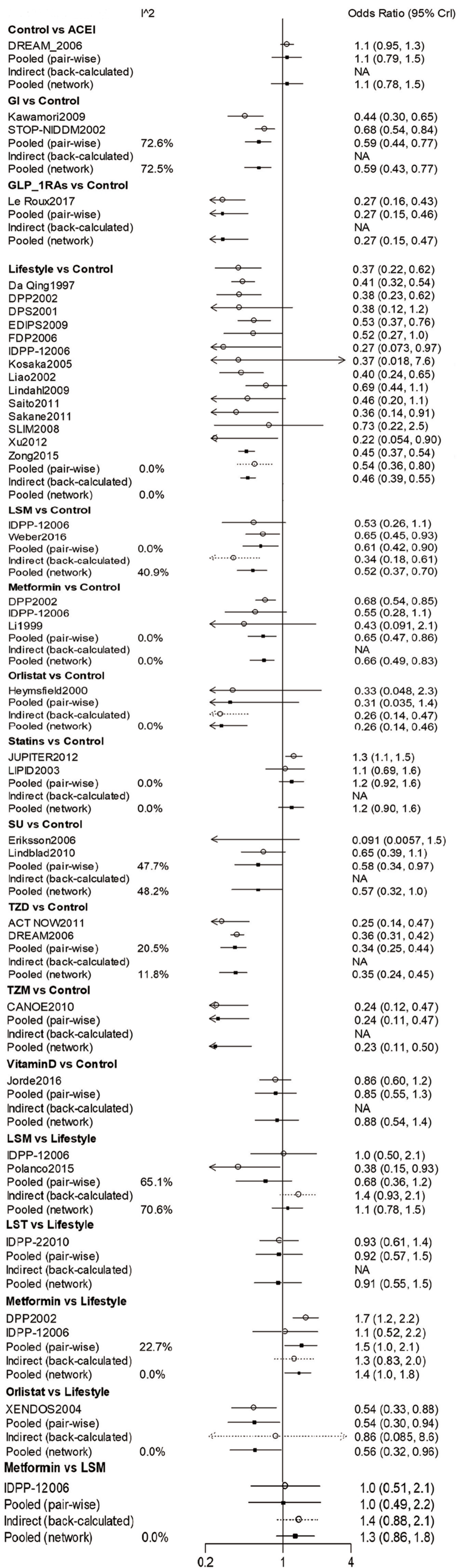

Supplementary Figure 1

Supplement: Supplementary Figure 1 — Heterogeneity test in network meta-analysis using I2 in pair-wise and network pooled comparison. [file Image_1.pdf]

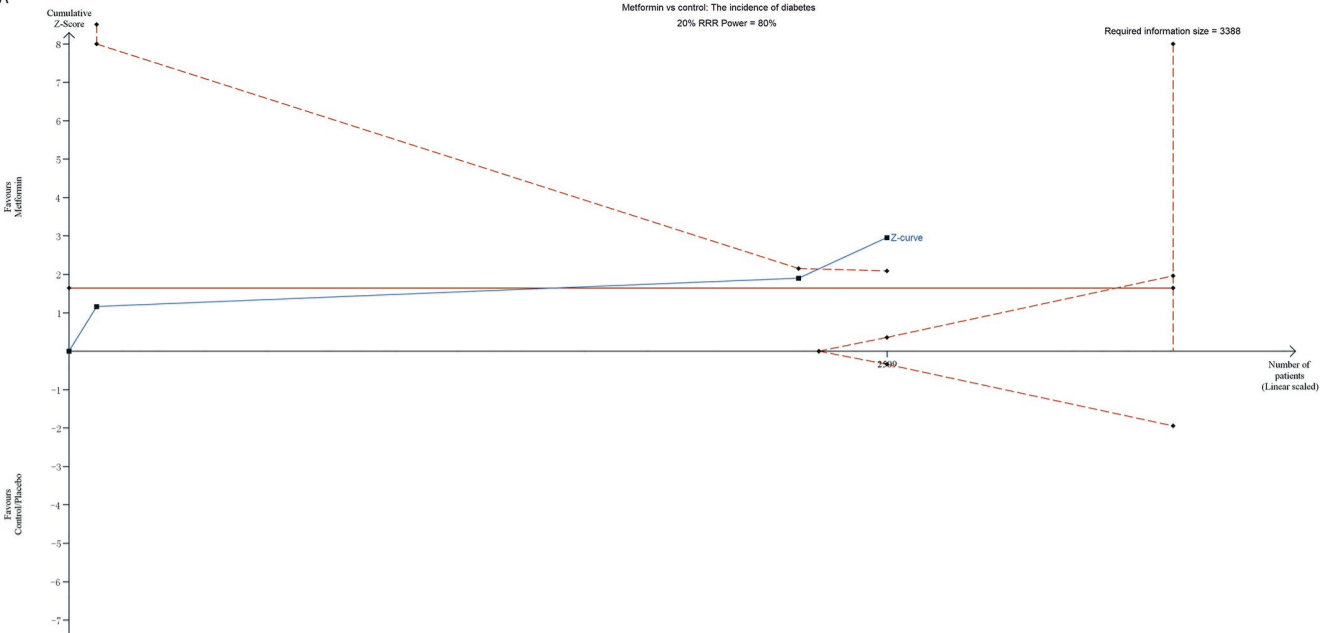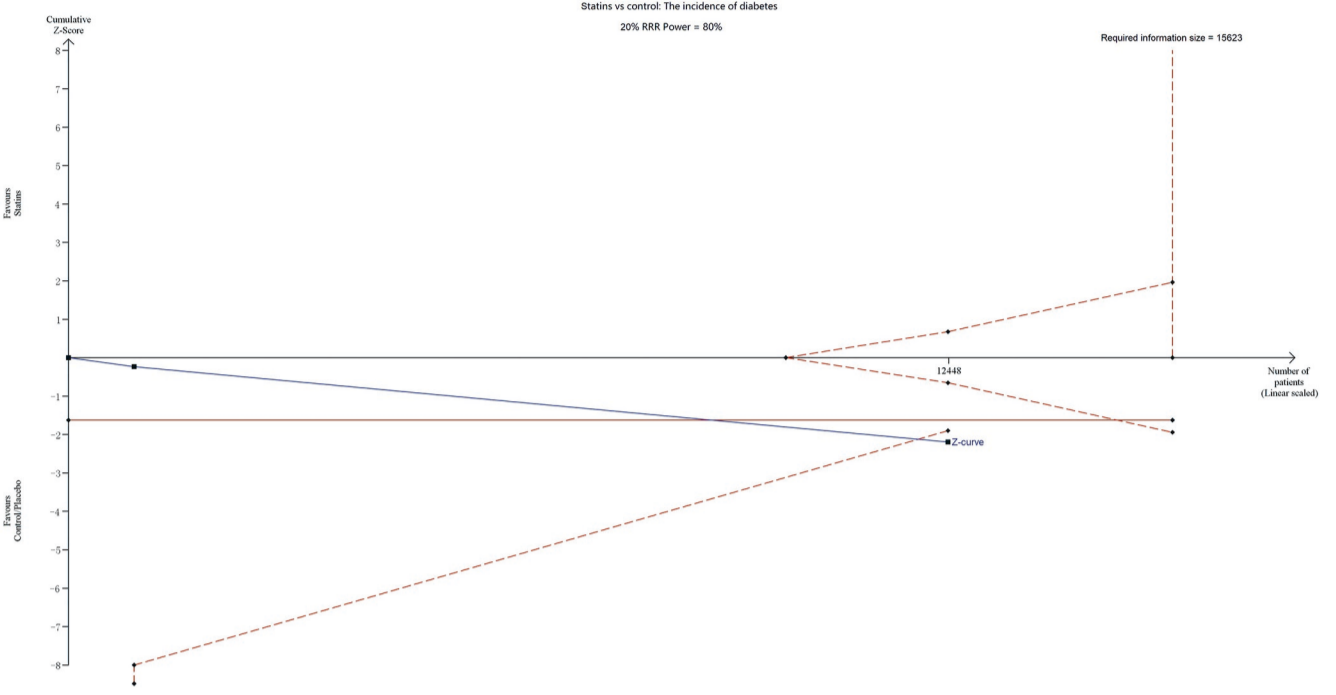

B

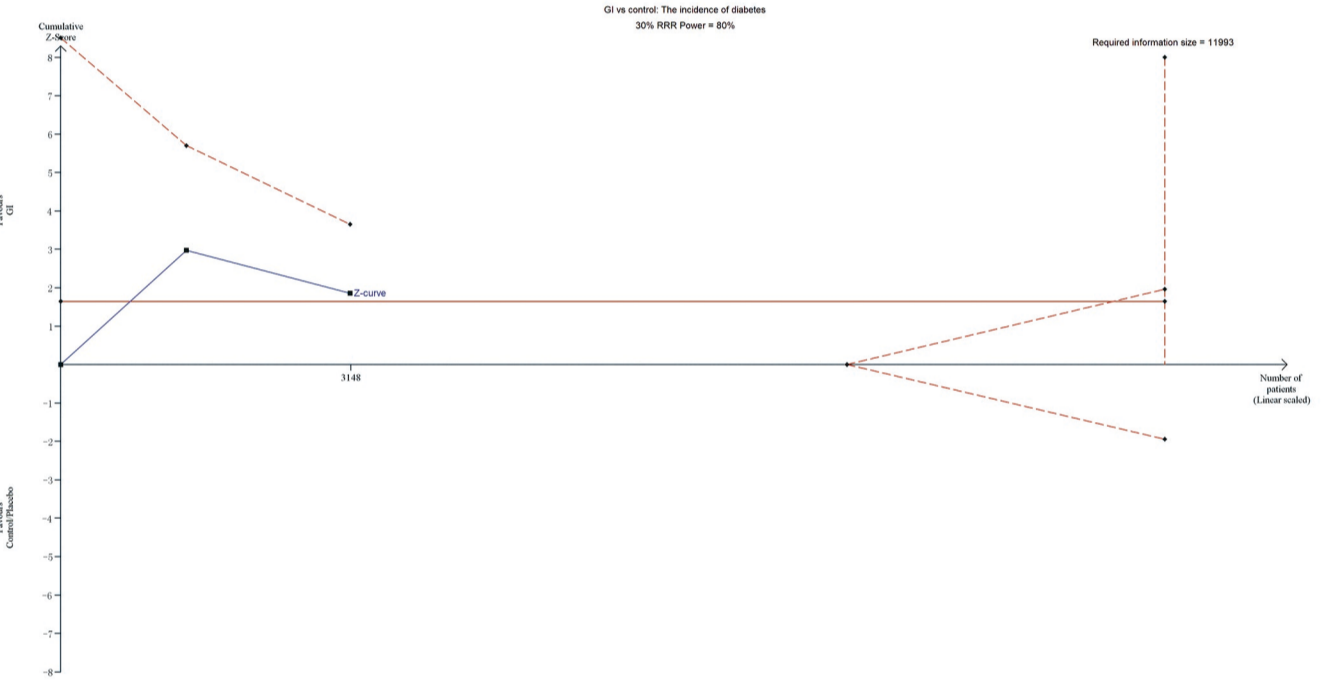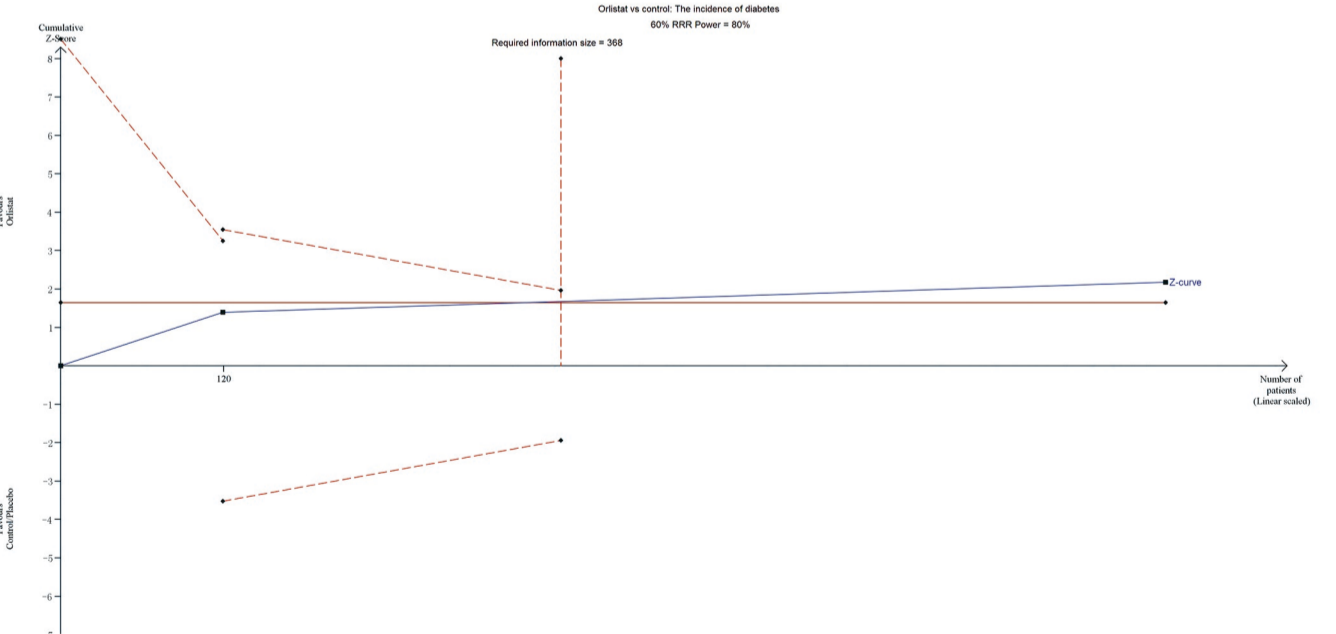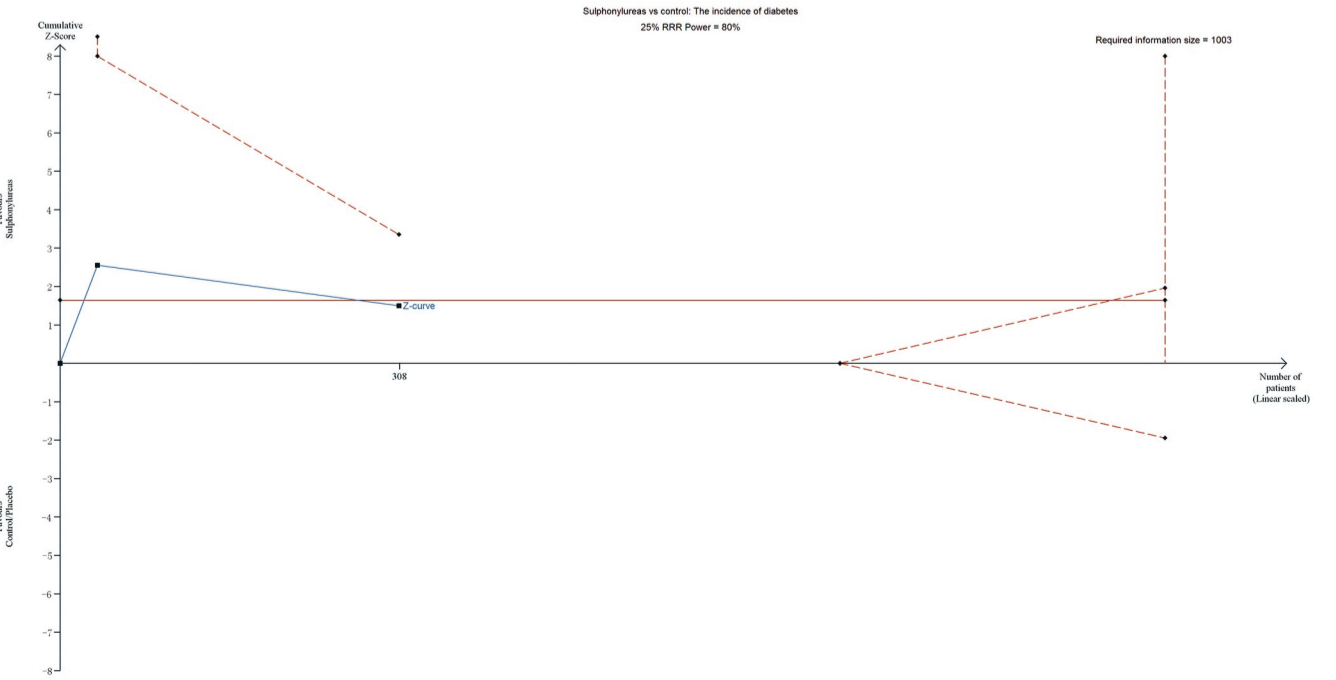

Supplementary Figure 2

Supplement: Supplementary Figure 2 — (A) TSA of metformin and statins. Effect of metformin vs. control on diabetes using a required information size of 3,388 participants in order to detect or reject a 20% RRR with a power of 80%; effect of statins vs. control on diabetes using a required information size of 15,632 participants in order to detect or reject a 20% relative increased risk with a power of 80%. (B) TSA of GI, orlistat and sulphonylureas. Effect of GI vs. control on diabetes using a required information size of 11,993 participants in order to detect or reject a 30% RRR with a power of 80%; effect of orlistat vs. control on diabetes using a required information size of 368 participants in order to detect or reject a 60% RRR with a power of 80%; effect of sulphonylureas vs. control on diabetes using a required information size of 1,003 participants in order to detect or reject a 25% RRR with a power of 80%. [file Image_2.pdf]
